# The Hering Memorial

**Published:** 1881-03

**Authors:** 


### THE HERING MEMORIAL.

[We publish the following appeal with great pleasure. The entire profession (and especially the Homœopathic College of Phila.) owes Dr. Hering an immense debt. They have now an opportunity of showing their gratitude. Therefore let them promptly and generously subscribe the necessary funds not only for this small volume, but also for publishing the unfinished works of our departed teacher. A small sum from each of the reported six thousand physicians will fulfill this duty. If this appeal be neglected the world will believe that our school has not only "departed from the strict inductive method of Hahnemann" but also from the commonest feelings of gratitude.]

*Philadelphia, January 1st*, 1881.

Dear Doctor,—At the "Hering Memorial Meeting" held in Philadelphia, on the tenth day of last October, at the same time similar Memorial Meetings were held in the chief cities of the United States and of Europe, it was unanimously resolved to collect the various speeches and eulogies delivered at these meetings into a volume, under the title of "The Hering Memorial," which should serve not only as an expression of the veneration and affection in which we hold the memory of our great colleague, but also as a monument to his surpassing excellence as a man and physician more enduring than any structure in bronze or stone, and one, which, we are sure, would be more in accord with his own wishes.

The undersigned, literary executors of Dr. Hering, were appointed to edit this Memorial volume for which the materials are already in hand, and are merely awaiting the necessary funds for publication.

The Rev. Dr. Furness has kindly consented to write a short Memoir of his old friend, and this, with the material before mentioned and various papers furnished by eminent physicians and by personal friends, will make a volume of several hundred pages which can not but prove of great professional and historical value, and at the same time its contents will be sufficiently varied, to prove attractive to general readers, even for the few minutes they are awaiting attention in the physician's offiice. The book will be handsomely bound and illustrated.

In order to accomplish this object, you are asked to send to any one of the undersigned, whatsoever sum you may find it a pleasure to give toward the publication of this book, in memory of one who gave freely of all he had to his beloved Homœopathy.

To all contributors to the publication fund, a copy of the book will be sent.

Messrs Boericke & Tafel, the well known publishers, have kindly consented to attend, without remuneration, to the distribution of the volumes; the artist furnishes the drawings as his contribution; there remains, therefore, as the sole expense of the book, the cost of paper, engraving, printing and binding. Whatever sum remains after paying these four items, will be presented to Mrs. Hering in the name of all the subscribers, of whose names a printed list will accompany each volume.

Yours respectfully,

C. G. Raue, M.D.
121 *North Tenth Street.*

C. B. Knerr, M.D.,
112 *North Twelfth Street.*

C. Mohr, M.D.,
555 *North Sixteenth Street.*

